# Synthesis and Evaluation of Compound Targeting α7 and β2 Subunits in Nicotinic Acetylcholinergic Receptor

**DOI:** 10.3390/molecules28248128

**Published:** 2023-12-16

**Authors:** Karanveer Singh, Allyson Ngo, Oshini V. Keerthisinghe, Krystal K. Patel, Christopher Liang, Jogeshwar Mukherjee

**Affiliations:** Preclinical Imaging, Department of Radiological Sciences, University of California-Irvine, Irvine, CA 92697, USA; karanves@uci.edu (K.S.); allyson1@uci.edu (A.N.); okeerthi@uci.edu (O.V.K.); kkpatel11@gmail.com (K.K.P.); liangc@uci.edu (C.L.)

**Keywords:** [^18^F]nifene, [^125^I]α-bungarotoxin, autoradiography, PET, mice, rat, amyloid plaques, α7β2 nAChR

## Abstract

Nicotinic acetylcholine receptors (nAChRs) are involved in various central nervous system functions and have also been implicated in several neurodegenerative disorders. The heteromeric α4β2* and homomeric α7 are two major nAChR subtypes which have been studied in the brain using positron emission tomography (PET). Our comparative autoradiographic studies of the two receptor types in the mouse and rat brains show major differences in the thalamus (α4β2* >> α7), hippocampus (α7 >> α4β2*), and subiculum (α4β2* >> α7). A relatively newer heteromeric α7β2 nAChR subtype has been identified in the brain which may have a greater role in neurodegeneration. We report the development of KS7 (3-(2-(*S*)-azetidinylmethoxy)-5-(1,4-diaza-bicyclo[3.2.2]nonane)pyridine) which incorporates structural features of Nifzetidine (high affinity for α4β2* nAChR) and ASEM (high affinity for α7 nAChR) in an effort to target α7 and β2 subunits in α7β2 nAChR. KS7 exhibited higher affinities (IC_50_ = 50 to 172 nM) for [^3^H]cytisine radiolabeled sites and weaker affinities (IC_50_ = 10 μM) for [^125^I]-α-bungarotoxin radiolabeled rat brain sites in several brain regions. The weaker affinity of KS7 to α7 nAChR may suggest lack of binding at the α7 subunit of α7β2 nAChR. A radiolabeled derivative of KS7 may be required to identify any specific binding to brain regions suggested to contain α7β2 nAChR.

## 1. Introduction

**Figure 1:**
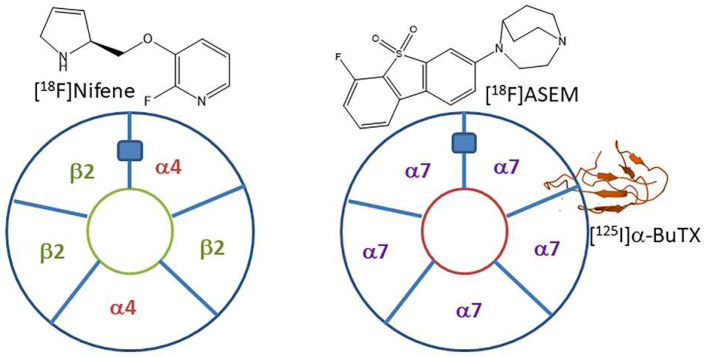
Neuronal nAChRs: Pentameric subunit layout of heteromeric α4_2_β2_3_ and homomeric α7_5_ nAChRs. The blue rectangle represents one of the acetylcholine sites. At this site, binding of PET radiotracers, [^18^F]nifene (α4β2*), and [^18^F]ASEM (α7) can occur. Also shown is α-[^125^I]BuTX, binding to homomeric α7_5_ nAChRs for autoradiography(structure of α-BuTX from RCSB PDB).

Neuronal nicotinic cholinergic receptors (nAChRs) are involved in learning, memory, addiction, and neuropsychatric illnesses [1]. Dysfunction of these receptors has been implicated in numerous human conditions including Alzheimer’s disease (AD) [2,3], Parkinson’s disease (PD) [4], and others. Several nAChR subtypes are present in the mammalian brain, with heteromeric α4β2 and homomeric α7 subtypes being in larger concentrations than the other subtypes ([5,6] Figure 1). Two molecules of acetylcholine (ACh) bind at the interface of α4 and β2 subunits in the α4β2 subtype, while five molecules of ACh bind at the α7-α7 interface in the α7 receptor subtype. Nicotine has a high affinity for the α4β2 subtypes (Ki = 5.45 nM) and is several orders of magnitude weaker for the α7 subtypes (Ki = 223 nM) [7].

Due to the clinical importance of nAChRs, evaluating the activity of these receptors can potentially assist in understanding human illness [8,9,10,11]. We have developed several positron emission tomography (PET) imaging agents for non-invasive imaging of high-affinity sites on α4β2* receptors using [^18^F]nifene [12,13,14,15] (Figure 1), [^18^F]nifrolene [16], and [^18^F]nifzetidine [17]; for single-photon emission computed tomography (SPECT), [^123^I]niodene was prepared [18]; and for PET/SPECT, Niofene [19] was examined. For α7 nAChRs, fewer in vivo PET imaging agents are available, with [^18^F]ASEM being the prominent one [20,21] (Figure 1). For in vitro studies of α7 nAChRs, [^125^I]α-iodobungarotoxin (α-[^125^I]BuTX) binds selectively and has been widely used [22,23,24].

Because cholinergic innervation may be affected in various pathophysiologies, PET and SPECT imaging studies of both α4β2* and α7 nAChRs have been underway. Human PET studies and SPECT studies have demonstrated that reduction in binding is implicated in the decline in executive function in AD [25,26]. More recent PET reports [8,9,27] in AD show a decrease in binding in several brain regions and a correlation with cognitive deficits with select brain regions. Results from the limited PET studies of α7 receptors have been mixed, suggesting an increase in mild cognitive impairment (MCI) and no change in AD [28]. Further studies are needed to ascertain the role of α7 receptors because of their higher concentration in the hippocampus, a region inflicted with AD pathology of Aβ plaques and neurofibrillary tangles [29].

More recently, heteromeric α7β2 nAChR subtype has been isolated and characterized in the mammalian forebrain [30,31,32,33]. Distribution of α7β2 subtype was found to be greater in the rodent basal forebrain cholinergic neurons and hippocampal interneurons and in human cerebral cortex neurons. However, the overall brain distribution and relative concentration of this new subtype (compared to α4β2 and α7 subtypes) is yet to be determined. In addition, functional properties of the α7β2 nAChR are yet to be fully understood. Compared to the α7 receptors, the α7β2 subtype displays greater sensitivity to oligomeric amyloid β peptide (Aβ) and this interaction between Aβ and α7β2 nAChR may be relevant in the pathogenesis of AD [2,34]. Since the α7β2 nAChR is pharmacologically distinct from the homomeric α7 nAChR, it likely plays a unique functional role in the mammalian brain. For these reasons, pursuit of a PET imaging agent for the α7β2 nAChR may be a worthwhile goal. However, considering the lower concentrations and structural similarities of the receptor subtypes, it remains to be determined if this a feasible target.

**Figure 2:**
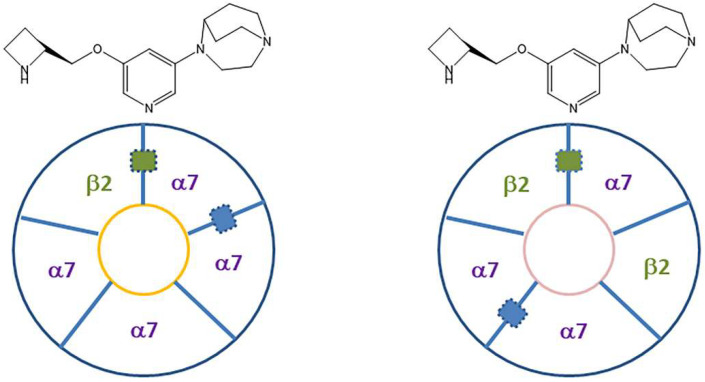
α7β2 Pentameric subunits layout: Heteromeric α7_4_β2_1_ and α7_3_β2_2_ nAChRs. The blue rectangle represents one of the potential α7-α7 interface binding sites. At this site, binding of KS7 may occur with the with the 1,4-diaza-bicyclo[3.2.2]nonane binding towards α7. It is suggested that β2 (green rectangle) may be a secondary binding site.

Two heteropentameric subtypes, α7_4_β2_1_ (α7-α7-α7-α7-β2) and α7_3_β2_2_ (α7-β2-α7-β2-α7), have been identified ([32]; Figure 2). No agonists or antagonists which bind to α7 subtype or α4β2 subtype have yet been identified to show selectivity for α7β2 subtype. It is not known if the ligand binding occurs at the α7-α7 interface or α7-β2 interface. Binding of α-[^125^I]BuTX occurs at the α7-α7 interface and therefore, it is unclear if it would bind to the α7-β2 interface [35]. It is also not known if α-[^125^I]BuTX would be able to identify the heteromeric α7_4_β2 and α7_3_β2_2_ nAChRs since they have the α7-α7 interfaces as well as α7-β2 interfaces.

**Figure 3:**
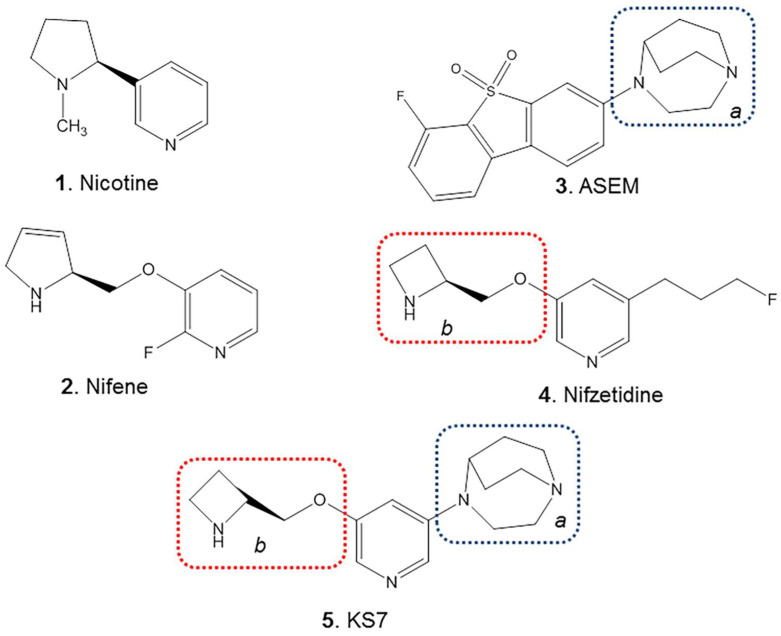
Chemical structures of PET Radiotracers: Heteromeric α4_2_β2_3_ (nicotine, nifene, nifzetidine) and homomeric α7_5_ (ASEM) nAChRs. Proposed structure (KS7) for heteromeric α7_4_β2 and α7_3_β2_2_ nAChRs incorporates the 1,4-diaza-bicyclo[3.2.2]nonane ring (blue dotted box) from ASEM (binds to α7) and azetidinylmethoxy ring (red dotted box) from Nifzetidine (binds to α4β2).

**Figure 4:**
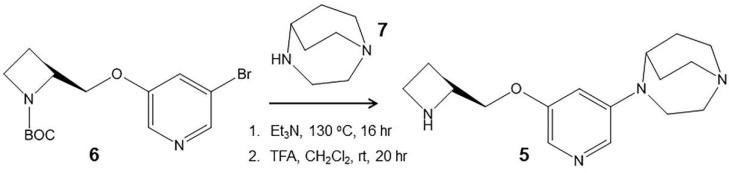
Synthesis: reaction scheme for synthesis of KS7 (**5**).

**Figure 5:**
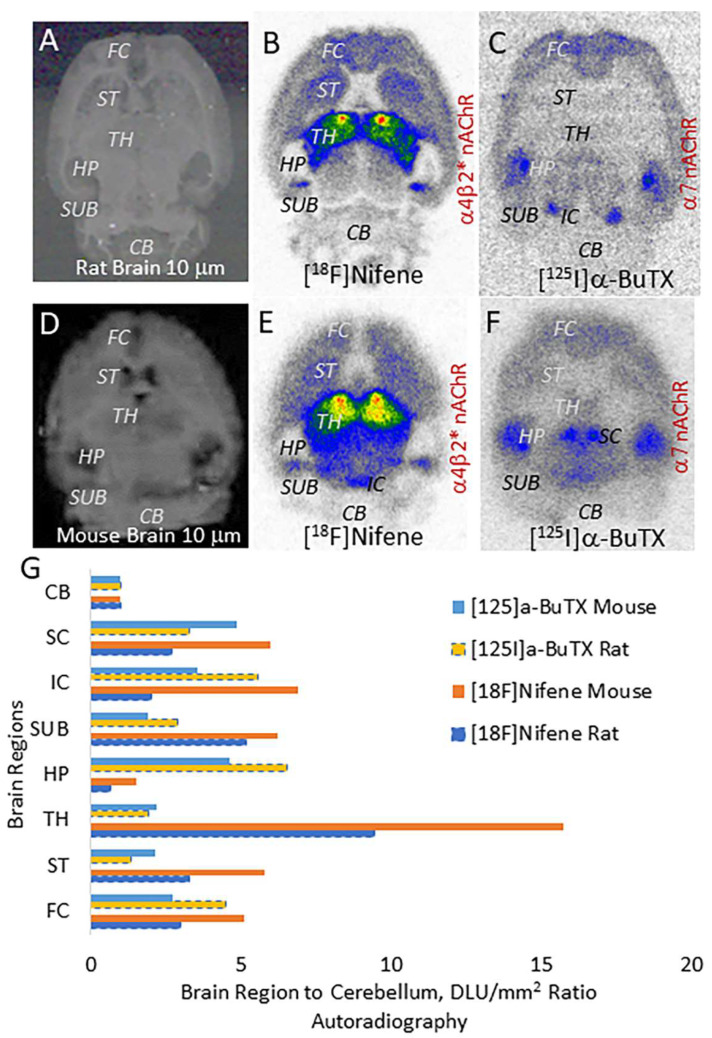
Autoradiography of α4β2 and α7 nAChRs: (**A**–**C**): Rat brain slices; (**A**) scan of 10 μm thick horizontal rat brain slice showing different regions; (**B**) binding of [^18^F]nifene to α4β2* nAChRs in the rat brain slice with highest binding in TH; (**C**) binding of α-[^125^I]BuTX to α7 nAChRs in rat brain slice with highest binding in HP. (**D**–**F**): Mouse brain slices; (**D**) scan of 10 μm thick horizontal mouse brain slice showing different regions; (**E**) binding of [^18^F]nifene to α4β2* nAChRs in mouse brain slice with highest binding in TH; (**F**) binding of α-[^125^I]BuTX to α7 nAChRs in mouse brain slice with highest binding in HP. (**G**) Comparison of ratios of brain regions to cerebellum in rat and mouse brain for [^18^F]nifene and α-[^125^I]BuTX (CB—cerebellum; SC—superior colliculus; IC—inferior colliculus; SUB—subiculum; HP—hippocampus; TH—thalamus; ST—striatum; FC—frontal cortex).

In an effort to identify a potential α7β2 ligand, we have chosen features of nifzetidine (Figure 3, **4**), a high affinity α4β2 ligand previously developed in our laboratories [17] and those of α7 agonists developed by Abbott laboratories [36] and ASEM ([20] Figure 3, **3**). The azetidine feature from nifzetidine which potentially binds to the β2 subunit and the 1,4-diaza-bicyclo[3.2.2]nonane feature from ASEM which potentially binds to the α7 subunit were both incorporated into KS7 (Figure 3, **5**) in an effort to bind to the α7-β2 interface.

Thus, in this paper, we report the following: (1) synthesis and characterization of 3-(2-(*S*)-azetidinylmethoxy)-5-(1,4-diaza-bicyclo[3.2.2]nonane)pyridine (KS7, Figure 4, **5**); (2) measurement of in vitro binding affinities of KS7 using [^3^H]cytisine to label the α4β2* nAChR sites; and (3) measurement of in vitro binding affinities KS7 in rat brain slices using α-[^125^I]BuTX to label the α7 nAChR sites. 

## 2. Results and Discussion

Distribution of α4β2* and α7 nAChRs, two major receptor subtypes in the mammalian brain have been well-studied. Our studies on the distribution of the two receptor subtypes, α4β2* and α7 nAChRs, in mice and rat brains show distinct areas of similarities and differences (Figure 5). Using [^18^F]nifene for α4β2* nAChRs, the ratio versus cerebellum (used as a reference region) for rat brains was in the order of thalamus > subiclum > striatum > frontal cortex > superior colliculus > inferior colliculus > hippocampus > cerebellum [12,13]. Similar binding profile was observed in the rat brains using [^3^H]cytisine [37]. The mice brains generally exhibited higher ratios compared to rats for [^18^F]nifene-labeled α4β2* nAChRs.

Using α-[^125^I]BuTX for α7 nAChRs, the ratio versus cerebellum (used as a reference region) for rat brains was in the order of hippocampus > inferior colliculus > frontal cortex > superior colliculus > subiculum > thalamus > striatum > cerebellum similar to previous reports for α7 nAChRs [21,24]. Some minor differences in the inferior and superior colliculi were observed between mouse and rat brains for α7 nAChRs.

These comparative autoradiographic studies of the two receptor types in the mouse and rat brains show major differences in the thalamus (α4β2* >> α7) and hippocampus (α7 >> α4β2*). Also noteworthy is the difference in subiculum (α4β2* >> α7). The colliculi (superior and inferior) exhibited presence of both α4β2* and α7 nAChRs, the latter appeared to be more discreet. The regional distribution of α-[^125^I]BuTX is comparable to the binding of the small molecule ASEM [21]. Similar to these findings in the rodents, human studies with α4β2* and α7 nAChRs PET radiotracers have been reported. In the case of α4β2* nAChRs, our comparative study of [^18^F]nifene across different species suggest a high degree of homology [7]. In the case of α7 nAChRs PET using [^18^F]ASEM, similarity across species has been reported [38].

In all species, there are a number of brain regions where there is some overlap between α7 and β2 nAChRs subunits [39,40,41]. Thus, heteromeric α7β2 nAChR subtype has been isolated and characterized and was found to be greater in the basal forebrain cholinergic neurons and hippocampal interneurons and in human cerebral cortex neurons [30,31,32,33]. Regional brain distribution and relative concentration of this new subtype is yet to be determined. The α7β2 subtype may be more sensitive to oligomeric Aβ peptide and this interaction between Aβ and α7β2 nAChR may be relevant in the pathogenesis of AD [2,34]. Since the heteromeric α7β2 nAChR is likely pharmacologically distinct from the homomeric α7 nAChR, a selective radioligand for imaging is necessary to study its distribution in the brain. Efforts have been made to assess [^125^I]α-BuTX binds to α7β2 nAChR [31,32]. Other α7 and α4β2* drugs have not been shown to clearly differentiate between α7 and α7β2 nAChRs [32]. For these reasons, pursuit of a drug compound for the α7β2 nAChR may be a worthwhile goal. If found selective, this drug could then be radiolabeled for imaging studies. Towards this goal, we designed and synthesized KS7 (Figure 3 and Figure 4) which is incorporated structural components of Nifzetidine (α4β2* nAChR) and ASEM (α7 nAChR). 

**Figure 6:**
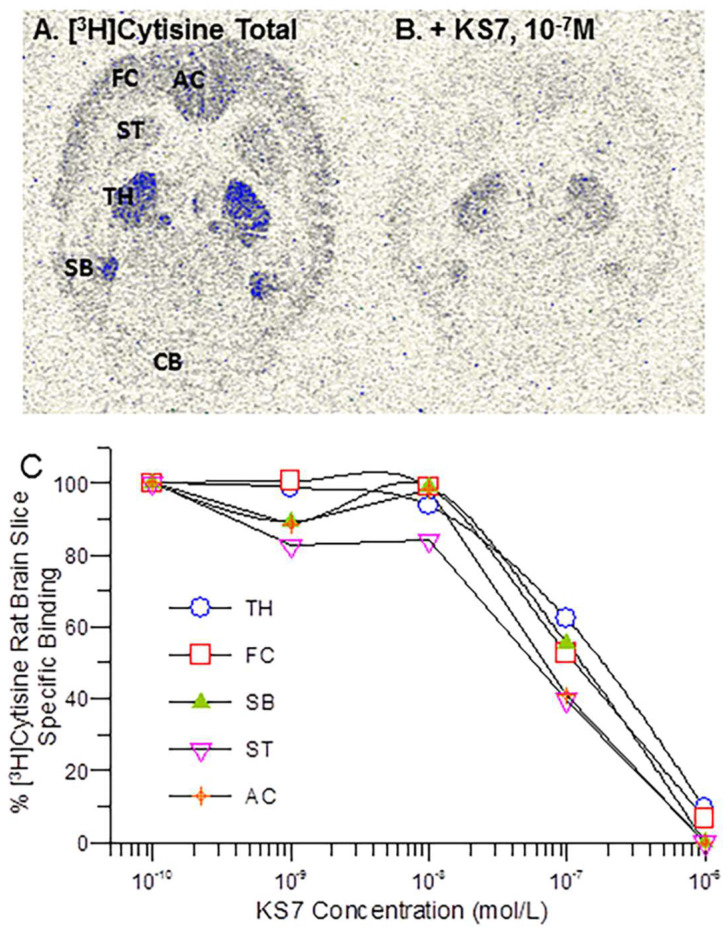
α4β2 Binding Affinity: Binding affinity of [^3^H]cytisine in the presence of KS7 on rat brain regions. (**A**) Total binding of [^3^H]cytisine in different brain regions (AC—anterior cingulate; FC—frontal cortex; ST—striatum; TH—thalamus; SB—subiculum; CB—cerebellum); (**B**) binding of rat brain slice at 10^−7^ M. (**C**) Competition binding curves of rat brain slices shown in (**A**,**B**).

In vitro binding affinity studies of KS7 were carried out on rat brain slices labeled with [^3^H]cytisine (Figure 6). Figure 6A shows [^3^H]cytisine labeling of rat brain regions of thalamus, frontal cortex, anterior cingulate, striatum, subiculum, and cerebellum as previously reported [37]. With increasing concentration of KS7, binding of [^3^H]cytisine was reduced from all brain regions. 

Measured inhibitory constants (IC_50_) of KS7 in the various brain regions were: striata = 86.9 nM; frontal cortex = 109 nM; thalamus = 134 nM; subiculum = 172 nM; anterior cingulate = 49.5 nM (Table 1).

**Figure 7:**
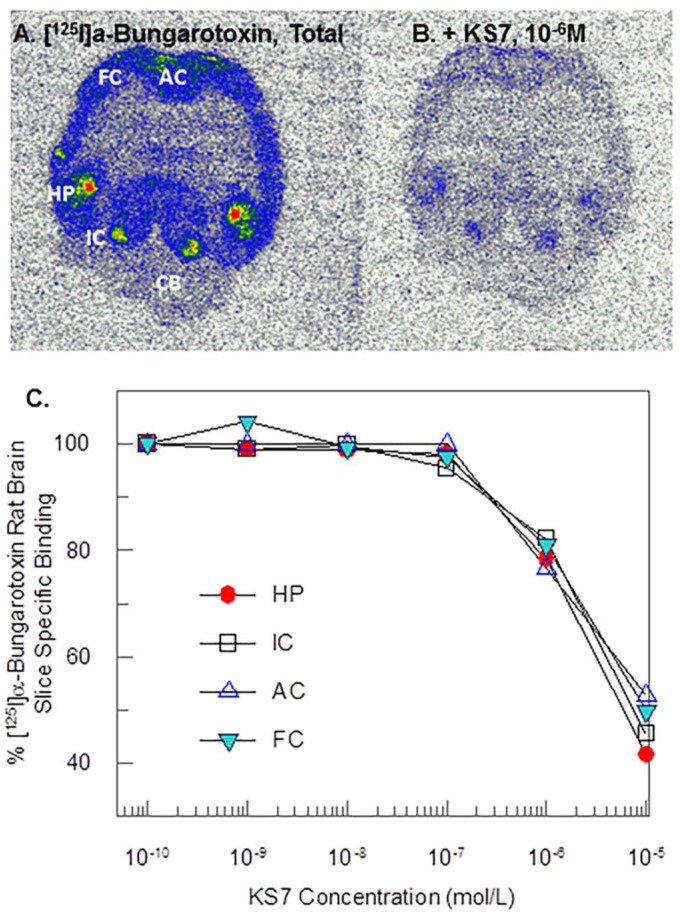
α7 Binding affinity: binding affinity of α-[^125^I]BuTX in the presence of KS7 on rat brain regions. (**A**) Total binding of α-[^125^I]BuTX in different brain regions (FC—frontal cortex; AC—anterior cingulate; HP—hippocampus; IC—inferior colliculus; CB—cerebellum); (**B**) binding of rat brain slice at 10^−6^ M. (**C**) Competition binding curves of rat brain slices shown in (**A**,**B**).

For α7 nAChRs, Figure 7 shows [^125^I]α-BuTX labeling of rat brain regions of frontal cortex, anterior cingulate, hippocampus, inferior colliculus, and cerebellum. With increasing concentration of KS7, binding of α-[^125^I]BuTX was reduced from all brain regions. Measured inhibitory constants (IC_50_) of KS7 in the various brain regions were frontal cortex = 9.9 μM; anterior cingulate = 12.6 μM; hippocampus = 33.2 μM; and inferior colliculus = 46.8 μM. The selectivity of KS in frontal cortex and anterior cingulate was in favor of α4β2* nAChR subtype.

**Figure 8:**
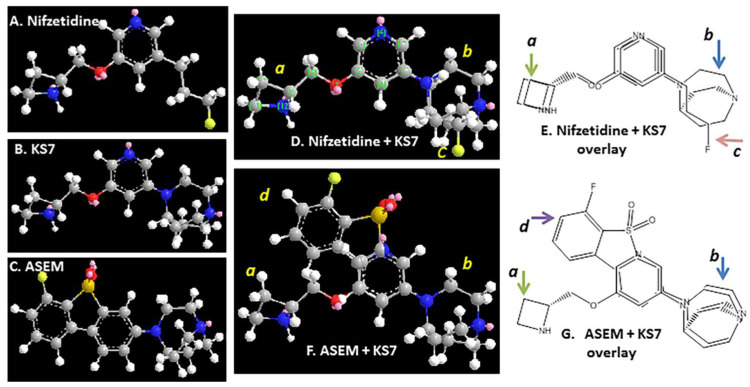
KS7 Structure comparisons: (**A**) minimized energy structure of nifzetidine with reported high affinity for α4β2* receptors; (**B**) minimized energy structure of KS7; (**C**) energy minimized structure of ASEM with reported high affinity for α7 nAChR. (**D**,**E**) Minimized energy structure and chemical structure of nifzetidine and KS7 overlay; (**F**,**G**) minimized energy structure and chemical structure of ASEM with KS7 overlay.

Based on the crystal structure of the α4β2* receptor [42], the high-affinity binding sites sit at the interface between the α4 and β2 subunits in the receptor complex. Binding of the azetidine ring nitrogen is likely at the α4 subunit side and since this part of the molecule remains the same in the case of KS7, moderate affinity for the α4β2* receptor sites is maintained. Figure 8 shows similarity of energy minimized structures of nifzeridine and KS7 in the overlay (Figure 8D,E). The presence of the azabicylononane ring may be causing a decrease in the affinity at the α4β2* receptor. Significant deviation in the overlay structures of ASEM and KS7 is seen (Figure 8F,G) which may be the reason for the larger decrease in the affinity at the α7 receptor sites. This may be favorable in terms of directing KS7 away from the α7 nAChRs.

Table 2 summarizes the affinities and selectivities for the various at the α4β2 and α7 nAChRs. The selectivity of KS7 seems to resemble nicotine, as a dual α4β2/α7 drug [43], but with significantly weaker affinities compared to nicotine.

## 3. Materials and Methods

### 3.1. General Methods

All chemicals and solvents were of analytical or HPLC grade from Aldrich Chemical Co. and Fisher Scientific. Electrospray mass spectra were obtained on a Model 7250 mass spectrometer (Micromass LCT, Milford, MA, USA). Proton NMR spectra were recorded on a Bruker OMEGA 600 MHz spectrometer. Analytical thin layer chromatography (TLC) was carried out on silica-coated plates (Baker-Flex, Phillipsburg, NJ, USA). Chromatographic separations were carried out on preparative TLC (silica gel GF 20 × 20 cm, 2 mm thick; Alltech Assoc. Inc., Deerfield, IL, USA) or silica gel flash columns or semi-preparative reverse-phase columns using the Gilson high-performance liquid chromatography (HPLC) systems. [^3^H]cytisine and α-[^125^I]BuTX, purchased from American Radiolabeled Chemicals, Inc., St Louis MO, were used for autoradiographic studies by exposing tissue samples on storage phosphor screens. The apposed phosphor screens were read and analyzed by OptiQuant acquisition and analysis program of the Cyclone Storage Phosphor System (Packard Instruments Co., Boston, MA, USA).

### 3.2. Animals

All animal studies were approved by the Institutional Animal Health Care and Use Committee (IACUC) of University of California-Irvine. Adult male Sprague Dawley rats (*n* = 4) were used in this study (280 g; 18–24 weeks). Rats were purchased from Jackson Laboratory. Adult male C57BL/6 mice were used in this study (28 g). Mice were purchased from Jackson Laboratory. All animals were housed under controlled temperatures of 22 °C ± 1 °C, in a 12 h light–dark cycle, on at 6:00 AM, with water and food chow ad libitum. Brains from the mice and rats were excised, and horizontal slices were prepared at 10 µm thickness using a Leica 1850 cryotome at −20 °C. The brain slices containing the cortex, striatum, thalamus, hippocampus, and cerebellum were stored in −80 °C freezer and used for binding studies.

### 3.3. Synthesis

(3-(2-(*S*)-azetidinylmethoxy)-5-(1,4-diazabicyclo[3.2.2]nonane)pyridine) (Figure 4, **5**; KS7): 3-Bromo-5-(1-*tert*-butoxycarbonyl-2-(*S*)-azetidinylmethoxy)pyridine (Figure 4, **6**) (34 mg; 0.1 mmol) was dissolved in triethylamine (0.1 mL). To this solution, 1,4-diaza-bicyclo[3.2.2]nonane (15 mg; 0.12 mmol) was added and the closed reaction 10 mL V-vial was heated at 130 °C for 16 h. The reaction mixture was purified on preparative silica gel TLC using 9:1 dichloromethane-methanol to provide of pure (3-(1-*tert*-butoxycarbonyl-2-(*S*)-azetidinylmethoxy)-5-(1,4-diazabicyclo[3.2.2]nonane)pyridine). Mass spectra (*m*/*z*, %): 389 ([M + H]^+^, 10%), 411 ([M + Na]^+^, 80%). We anticipated this reaction to be sluggish and low-yielding (due to the 3-bromo position in the pyridine ring). The *N*-BOC intermediate was obtained in <5% yield. Prolonged reaction time was not useful in increasing the yield. Deprotection of the N-*tert*-butoxycarbonyl was carried out by treatment with trifluoroacetic acid (TFA; 0.1 mL) in dichloromethane (1 mL) at room temperature or 24 h. The final product, (3-(2-(*S*)-azetidinylmethoxy)-5-(1,4-diazabicyclo[3.2.2]-nonane)pyridine) (Figure 4, **5**, KS7) was obtained by preparative TLC (1:1 dichloromethane-methanol) in <5% overall yield. Removal of the *N*-BOC protecting group using trifluoroacetic acid resulted in KS7 which was isolated as a KS7 trifluoroacetate salt was used for in vitro studies. Mass spectra (*m*/*z*, %): 312 ([M + Na]^+^, 100%); 1H NMR (CDCl_3_, 600 MHz) δ ppm: 8.28 (m, 2H), 7.43 (dd, J = 1.8, 2.6 Hz, 1H), 4.33 (m, 1H), 4.13 (m, 4H), 3.89 (m, 2H), 3.60 (m, 2H), 3.20 (m, 4H), 2.95 (m, 2H), 2.33 (m, 2H), 2.20 (m, 2H), 1.95 (m, 2H).

### 3.4. In Vitro Autoradiographic Studies α4β2* nAChR—[^18^F]Nifene

Autoradiography of [^18^F]nifene in mice and rat brain slices were previously reported [3,12]. Briefly, slides with brain slices were incubated with [^18^F]nifene in a Tris buffer pH 7.4 (60 mL; 37 kBq/mL) and was added to the chambers and were incubated at 25 °C for 1 h. Nonspecific binding was measured in separate chambers in the presence of 300 μM nicotine. The slices were then washed with cold buffer twice, 3 min each time, Tris buffer, and cold water for rinse. The brain sections were air dried, exposed overnight on a phosphor film, and then placed on the Phosphor Autoradiographic Imaging System/Cyclone Storage Phosphor System (Packard Instruments Co.). Regions of interest (ROIs) were drawn on the slices and the extent of binding of [^18^F]nifene was measured in Digital Light Units/mm^2^ (DLU/mm^2^) using the OptiQuant acquisition and analysis program (Packard Instruments Co.).

### 3.5. In Vitro Binding Affinity Studies α4β2* nAChR—[^3^H]Cytisine

Our previously published procedures were used for [^3^H]cytisine experiments [37]. Horizontal brain slices, 10 µm thick from male Sprague Dawley rats, were preincubated in the buffer (50 mmol/L Tris HCl containing 120 mmol/L NaCl, 5 mmol/L KCl, 2.5 mmol/L CaCl_2_, 1 mmol/L MgCl_2_, pH 7.4) for 10 min. The pre-incubation buffer was then discarded and the slices were incubated with [^3^H]cytisine (2.5 nM, specific activity of 32.7 Ci/mmol) at 2 °C for 75 min. Binding was measured in the presence of 0.1 nM to 1 µM of KS7 and 300 µM of nicotine was used for nonspecific binding. After incubation, slices were washed twice (2 min each wash) with ice-cold Tris buffer, pH 7.4, followed by a quick rinse in cold (0–5 °C) deionized water. The slides were then air dried and the radiolabeled brain sections were apposed on storage phosphor screens for two weeks (Perkin Elmer Multisensitive, Medium MS, New Jersy, USA). For autoradiographic analysis, the apposed phosphor screens were read and analyzed by OptiQuant acquisition and analysis program of the Cyclone Storage Phosphor System (Packard Instruments Co., Boston, MA, USA). Regions of interest of same size were drawn and analyzed on brain regions using OptiQuant software version 2 and binding of [^3^H]cytisine measured in DLU/mm^2^. Data were analyzed using following procedure: (a) the non-specific binding of [^3^H]cytisine was subtracted for all samples; (b) the specific binding was normalized to 100% (no competitive ligand); and (c) the binding isotherms were fit to the Hill equation (KELL BioSoft software (v 6), Cambridge, UK).

### 3.6. In Vitro Autoradiographic Studies α7 nAChR—α-[^125^I] BuTX

Autoradiography of α-[^125^I]BuTX in mice and rat brain slices were previously reported [21,24]. The horizontal brain slices were preincubated in 50 mM Tris HCl, pH 7.3, containing 0.1% bovine serum albumin (BSA) at room temperature for 30 min. After the preincubation, the slices were incubated with α-[^125^I]BuTX (0.2 nM, specific activity of 2200 Ci/mmol) at room temperature for 120 min. Nonspecific binding was measured in separate chambers in the presence of 300 μM nicotine. After incubation, slices were washed three times (10 min each wash) with ice-cold Tris buffer, pH 7.3, followed by a quick rinse in cold (0–5 °C) deionized water. The brain sections were air dried, exposed for a week on a phosphor film, and then placed on the Phosphor Autoradiographic Imaging System/Cyclone Storage Phosphor System (Packard Instruments Co.). Regions of interest (ROIs) were drawn on the slices and the extent of binding of [^125^I]α-bungarotoxin was measured in DLU/mm^2^ using the OptiQuant acquisition and analysis program (Packard Instruments Co.).

### 3.7. In Vitro Binding Affinity Studies α7 nAChR—[^125^I]α-Bungarotoxin

For the α-[^125^I]BuTX assay, the reported method was used [24]. The horizontal rat brain slices were preincubated in 50 mM Tris HCl, pH 7.3, containing 0.1% bovine serum albumin (BSA) at room temperature for 30 min. The pre-incubation buffer was then discarded and the slices were incubated with α-[^125^I]BuTX (0.2 nM, specific activity of 2200 Ci/mmol) at room temperature for 120 min. Binding was measured in the presence of 0.1 nM to 1 µM of KS7 and 300 µM of nicotine was used for nonspecific binding. After incubation, slices were washed three times (10 min each wash) with ice-cold Tris buffer, pH 7.3, followed by a quick rinse in cold (0–5 °C) deionized water. The slides were then air dried and the radiolabeled brain sections were apposed on storage phosphor screens for one week (Perkin Elmer Multisensitive, Medium MS). For autoradiographic analysis, the apposed phosphor screens were read and analyzed by OptiQuant acquisition and analysis program of the Cyclone Storage Phosphor System (Packard Instruments Co., Boston, MA, USA).

Regions of interest of same size were drawn and analyzed on brain regions using OptiQuant software and binding of α-[^125^I]BuTX measured in digital light units/mm^2^ (DLU/mm^2^). Data were analyzed using following procedure: (a) the non-specific binding of α-[^125^I] BuTX was subtracted for all samples; (b) the specific binding was normalized to 100% (no competitive ligand); and (c) the binding isotherms were fit to the Hill equation (KELL BioSoft software (v 6), Cambridge, UK).

## 4. Conclusions

Heteromeric α4β2 and homomeric α7 nAChR subtypes have well-defined brain distribution patterns across the various species. A relatively new heteromeric α7β2 nAChR has been identified for which there is currently no selective drug or imaging agent. A new compound, KS7 has been synthesized as a potential candidate that may bind to the α7 and β2 subunits. The moderate to weak affinities of KS7 to α4β2 and α7 sites is promising. Cell lines expressing the α7β2 nAChRs will be necessary to assess selectivity in binding. A radiolabeled derivative of KS7 using fluorine-18 or carbon-11 labeling either directly on the pyridine ring or other strategies is planned [12,16,49,50]. This may enable autoradiographic studies to identify any discreet binding to hippocampal regions or the forebrain regions, where α7β2 nAChRs may be present. Imaging studies related to the association of Aβ plaque with nAChR subtypes will provide useful information on the potential role of this interaction in AD transgenic mice models [3,51].

## Figures and Tables

**Table 1:** Inhibition constants (IC_50_) for KS7 at nicotinic receptors.

Brain Region	[^3^H]Cytisine, α4β2* nAChRs	[^125^I]α-BuTX, α7* nAChRs	α7/α4β2 Ratio
Thalamus	1.34 × 10^−7^	-	-
Frontal Cortex	1.09 × 10^−7^	9.90 × 10^−6^	91
Anterior Cingulate	4.95 × 10^−8^	1.26 × 10^−5^	254
Subiculum	1.72 × 10^−7^	-	-
Striatum	8.69 × 10^−8^	-	-
Hippocampus	-	3.32 × 10^−5^	-
Inferior Colliculus	-	4.68 × 10^−5^	-

**Table 2:** Binding affinities of various drugs for α4β2 and α7 nAChRs.

Drug	α4β2*, nM	α7*, nM	α7/α4β2 Ratio	α4β2/α7 Ratio
Nicotine	6.93 ^a^	223 ^c^	32	0.03
	1.68 ^b^		133	
Nifene	1.07 ^a^	169 ^c^	158	0.007
	0.50 ^b^		338	
Cytisine	1.51 ^d^	691 ^e^	544 ^e^	0.002
	1.27 ^e^			
α-Iodobungarotoxin	>1000 ^f^	0.50 ^g^	0.0005	2000
IodoASEM	1707 ^h^	0.5 ^h^	0.0003	3414 ^h^
ASEM	562 ^i^	0.37 ^i^	0.0007	1370 ^i^
KS7	109 ^j^	9900 ^j^	91 ^j^	0.01 ^j^

^a^ Rat brain cortex labeled with ^3^H-epibatidine [7]. ^b^ Rat brain homogenates labeled with ^3^H-cytisine [12]. ^c^ Rat cloned receptors labeled with α-[^125^I]BuTX [7]. ^d^ Cytisine tested in rat [44]; ^e^ Cytisine tested on heterologously expressed human receptors; α4β2 in HEK293 cells and α7 nAChR in SH-SY5Y human neuroblastoma cells [45]. ^f^ [^3^H]nicotine inhibition by α-bungarotoxin [46]; ^g^ α-iodobungarotoxin tested in rat [41]; ^h^ Iodo-ASEM affinities HEK293 membranes expressing α7 and α4β2 nAChRs [47]; ^i^ HEK293 cells transfected with α7 and α4β2 nAChRs [48]; ^j^ Frontal cortex values for KS7 taken from Table 1.

## Data Availability

The data that support the findings of this study are available from the corresponding author upon reasonable request.
